# The role of peripapillary vessel density and retinal nerve fiber layer thickness in diagnosing and monitoring myopic glaucoma

**DOI:** 10.3389/fmed.2025.1620968

**Published:** 2025-06-16

**Authors:** Qin Chen, Qingwei Meng, Yijin Tao, Jing Liu, Zhu Zeng, Ye Sheng, Wenyan Yang, Qing Cun, Xiaojun Lin, Xi Chen, Guangkun Huang, Hua Zhong

**Affiliations:** ^1^Department of Ophthalmology, The First Affiliated Hospital with Nanjing Medical University, Nanjing, China; ^2^Department of Ophthalmology, The First Affiliated Hospital of Kunming Medical University, Kunming, China; ^3^Department of Ophthalmology, HeiLongJiang Red Cross Sengong General Hospital, Harbin, China

**Keywords:** high myopia, glaucoma, peripapillary vessel density (pVD), peripapillary retinal nerve fiber layer (pRNFL), visual field mean sensitivity (VFMS)

## Abstract

**Objective:**

To investigate changes in peripapillary vessel density (pVD) and retinal nerve fiber layer thickness (pRNFL) in highly myopic glaucoma patients compared to non-highly myopic glaucoma patients and healthy controls, and to evaluate their diagnostic capabilities using optical coherence tomography angiography (OCTA), and to explore the relationship between these biomarkers and visual function.

**Methods:**

A total of 382 eyes were recruited, including 101 highly myopic glaucoma eyes, 101 highly myopic control eyes, 90 non - highly myopic glaucoma eyes, and 90 non - highly myopic control eyes. Propensity score matching (PSM) was applied to balance age and axial length between groups. All subjects received comprehensive ophthalmic examinations. OCTA was used to measure pVD, spectral - domain OCT (SD - OCT) for pRNFL, and Humphrey 30–2 SITA standard visual field (VF) testing was performed. pVD and pRNFL were measured across eight peripapillary sectors. Pearson correlation and linear regression analyses were used to assess the relationships between pVD, pRNFL, and visual field mean sensitivity (VFMS). Receiver operating characteristic (ROC) curve analyses were carried out to evaluate the diagnostic performance.

**Results:**

Both highly myopic glaucoma and non-highly myopic glaucoma groups exhibited significantly lower pVD, pRNFL, and VFMS compared to their respective controls (*p* < 0.001). In highly myopic glaucoma, average pVD was 37.66% versus 46.40% in controls (*p* < 0.001), and pRNFL was 71.13 μm versus 101.22 μm in controls (*p* < 0.001). pVD showed stronger correlations with VFMS than pRNFL in both glaucoma groups (highly myopic: *r* = 0.681 vs. *r* = 0.504; non-highly myopic: *r* = 0.749 vs. *r* = 0.722; *p* < 0.001). ROC analysis demonstrated that the pRNFL and pVD have comparable diagnostic abilities in the early-stage of glaucoma (*p* > 0.05). However, the pRNFL outperforms the pVD in average diagnostic ability (*p <* 0.05). Combining superior-temporal (ST) and inferior-temporal (IT) regions achieved the highest diagnostic accuracy (AUC_pVD_: 0.905 and 0.965; AUC_pRNFL_: 0.934 and 0.942) for both glaucoma groups.

**Conclusion:**

pVD and pRNFL are valuable diagnostic biomarkers for myopic glaucoma. pVD demonstrated a stronger correlation with visual function, making it a promising tool for early glaucoma diagnosis and monitoring in highly myopic patients. Integrating pVD with pRNFL enhances diagnostic precision, particularly in highly myopic patients.

## Introduction

Myopia is a highly prevalent ocular condition and an independent risk factor for glaucoma, particularly in highly myopic eyes (1, 2). Diagnosing glaucoma in myopic eyes is complicated by structural changes in the retina and optic disc caused by myopia, including optic disc tilt, rotation, focal lamina cribrosa defects, and peripapillary atrophy (3). Furthermore, visual field impairments associated with high myopia can mimic glaucomatous damage, making differentiation between the two conditions difficult, especially in early glaucoma stages. Consequently, precise diagnosis and continuous monitoring of glaucoma in myopic eyes are essential.

Retinal nerve fiber layer (RNFL) thinning is a hallmark of glaucomatous optic nerve damage. However, assessing RNFL using optical coherence tomography (OCT) in highly myopic individuals is limited by factors such as axial length elongation, peripapillary atrophy, and segmentation errors (4). In advanced glaucoma cases, the RNFL often reaches a floor effect, further complicating disease progression tracking (5).

Optical coherence tomography angiography (OCTA) has emerged as a valuable tool for assessing both glaucoma and myopia due to its noninvasive nature and ability to provide quantitative analysis of the retinal and optic disc regions, including peripapillary vessel density (pVD) (6). The pVD, which supplies the nerve fiber layer on the optic disc surface (7), is particularly vulnerable to hemodynamic disturbances and cause damage to the optic nerve (8). Recent studies indicate that pVD is reduced in glaucoma and high myopia (9), and its reduction correlates with visual field (VF) damage severity, independent of structural loss (10). These findings suggest that pVD could serve as a diagnostic marker for glaucoma in myopic patients, although some evidence indicates that RNFL may have stronger diagnostic capabilities in such cases (11, 12). The interplay between blood flow and structural changes in glaucoma with high myopia remains complex and poorly understood.

To date, there has been no quantitative analysis of global and regional peripapillary microvasculature in highly myopic glaucoma patients compared to healthy controls. In this study, propensity score matching (PSM) was employed to match the highly myopic glaucoma group with healthy controls based on age and axial length, enabling more accurate comparisons of the diagnostic roles of RNFL thickness and pVD. The objectives of this research include the assessment of the diagnostic utility of pVD parameters, identifying the most sensitive diagnostic regions, and examining the relationship between pVD and visual function across different stages of glaucoma in highly myopic patients. By clarifying the roles of pVD and RNFL, this research aims to enhance the diagnosis and monitoring of glaucomatous visual field damage in highly myopic eyes.

## Materials and methods

### Subjects

**Figure 1:**
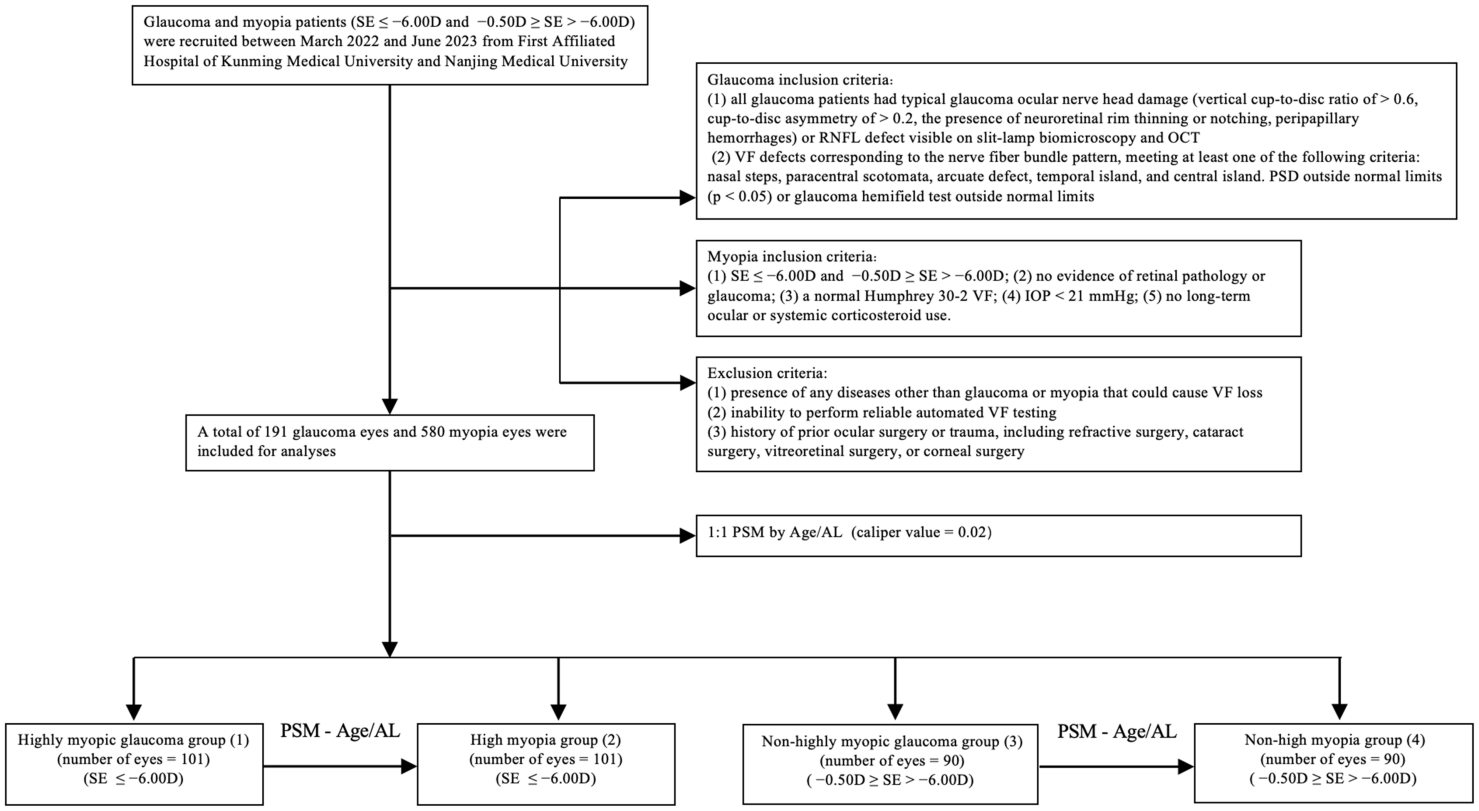
Flowchart depicting recruitment of participants in the study.

This multicenter cross-sectional study was conducted at the First Affiliated Hospital of Kunming Medical University and Nanjing Medical University, China. Glaucoma and myopia patients were recruited between March 2022 and June 2023. The Institutional Review Board of Kunming Medical University and the First Affiliated Hospital of Nanjing Medical University approved the study. Informed consent was obtained from all the participants. And Ethics Committee approval was obtained. This project was conducted in line with the principles of the Helsinki Declaration. A flowchart illustrating the case–control matching process is shown in Figure 1.

All subjects underwent complete ophthalmic examinations, including slit-lamp biomicroscopy, best-corrected visual acuity (BCVA), intraocular pressure (IOP), gonioscopy, axial length (AL) (IOL Master 700; Carl Zeiss Meditec, Dublin, California, USA) and central corneal thickness (CCT; DGH-550; DGH Technology, Exton, Pennsylvania, USA), and standard automated perimetry (Carl Zeiss Meditec, Dublin, CA, USA). Peripapillary RNFL thickness was measured by spectral-domain (SD) OCT (RTVue, XR, Avanti, Optuovue, Inc., Fremont, CA, USA), and peripapillary VD was obtained by OCTA imaging (RTVue, XR, Avanti, Optuovue, Inc., Fremont, CA, USA).

The inclusion criteria for the glaucoma group were: (1) all glaucoma patients had typical glaucoma ocular nerve head damage (vertical cup-to-disc ratio of > 0.6, cup-to-disc asymmetry of > 0.2, the presence of neuroretinal rim thinning or notching, peripapillary hemorrhages) or RNFL defect visible on slit-lamp biomicroscopy and OCT; and (2) VF defects corresponding to the nerve fiber bundle pattern, meeting at least one of the following criteria: nasal steps, paracentral scotomata, arcuate defect, temporal island, and central island. Pattern standard deviation (PSD) outside normal limits (*p* < 0.05) or glaucoma hemifield test outside normal limits.

For the control group, the inclusion criteria were: (1) spherical equivalent (SE) ≤ −6.00D and −0.50D ≥ SE > −6.00D; (2) no evidence of retinal pathology or glaucoma; (3) a normal Humphrey 30–2 VF; (4) intraocular pressure < 21 mmHg; (5) no long-term ocular or systemic corticosteroid use.

The exclusion criteria for glaucoma and myopia groups were: (1) presence of any diseases other than glaucoma or myopia that could cause VF loss; (2) inability to perform reliable automated VF testing; (3) history of prior ocular surgery or trauma, including refractive surgery, cataract surgery, vitreoretinal surgery, or corneal surgery.

### OCT imaging

RNFL imaging was performed with the SD-OCT device (RTVue, XR, Avanti, Optuovue, Inc., Fremont, CA, USA) using a scanning laser diode to emit a scan beam with a wavelength of 840 nm to provide images of ocular microstructures (13). Using the 4.5-mm diameter RTVue protocols that calculate the peripapillary images of nerve fiber layer thickness from ILM to NFL layer. The peripapillary RNFL thickness parameters were automatically calculated by the SD-OCT and divided into eight regions. Criteria for determining scan quality included a signal strength of 30 or higher for RTVue, a clear fundus image where the optic disc and scan circle were visible both before and during image acquisition, and the absence of en-face OCT image distortions caused by blinking or eye movements. Additionally, OCT scans with algorithmic failures in retinal layer segmentation that could not be corrected manually were excluded.

### OCTA imaging

**Figure 2:**
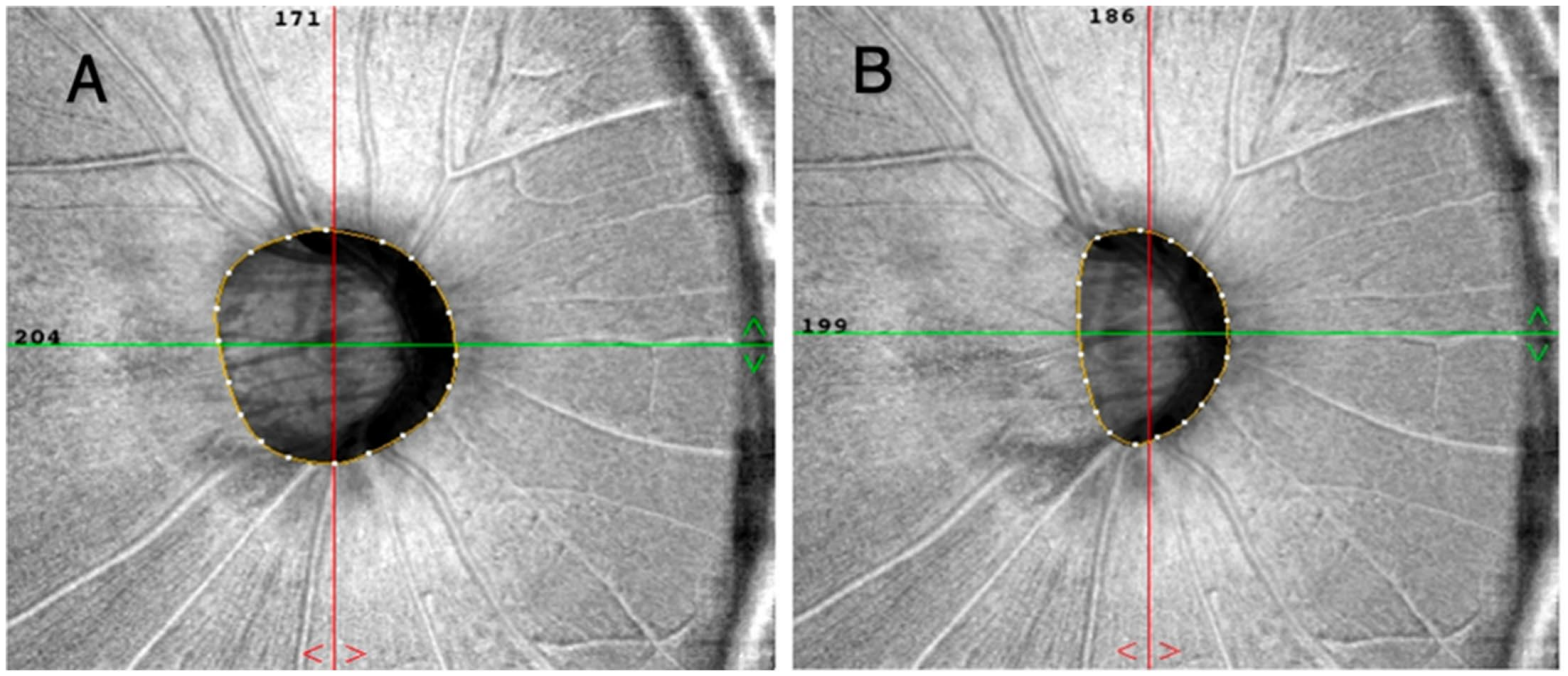
The boundaries of the optic disc were automatically and manually delineated at **(A,B)**. The center of the optic disc was determined by automatically fitting the best ellipse to the boundary.

The OCT-A images were acquired utilizing a commercially available spectral-domain OCT device (RTVue, XR, Avanti; Optuovue, Inc., Fremont, CA, USA). This system operates at a scan rate of 70,000 A-scans per second, with the scan beam wavelength set at a central value of 840 nm and a bandwidth of 45 nm. The cube scanning protocol involved 73 B-scans, each composed of 768 A-scans, and these scans were captured within a 4.5 × 4.5 mm squared region centered on the optic nerve head (ONH). The capillary densities from the internal limiting membrane (ILM) to the posterior boundary of the retinal nerve fiber layer (RNFL) were visualized using the standard AngioVue software, and the resultant measurement was referred to as the RPC density image. For the quantification of capillary density, the built-in large-vessel masking function was employed. This function eliminates vessels with a diameter of ≥ 3 pixels. In the context of 4.5-mm AngioVue optic disc (AngioDisc) scans (Optuovue 2018.1.1.63 software version), a 3-pixel diameter roughly corresponds to a physical dimension of ≥ 33 μm (14). The peripapillary region was measured at a 1-mm-wide elliptical annulus extending outward from the optic disc boundary (15). The vessel density (VD) was defined as the percentage area occupied by microvasculature, and the central 2 mm diameter circle centered on the optic disc based on the en-face reflectance image was excluded in the 4.5 × 4.5-mm scan area (16). Moreover, the boundary of the optic disc was manually delineated for patients with high myopia. The center of the optic disc was determined by automatically finding the best ellipse fit of the optic disc boundary (Figure 2). The OCTA images with a resolution of 304 × 304 pixels were processed using the Split-Spectrum Amplitude Decorrelation Angiography (SSADA) algorithm to enhance their visualization. Images were rechecked or excluded if they had (1) scan quality < 7; (2) poor clarity; (3) motion artifacts; (4) residual motion artifacts visible as irregular vessel patterns on the en face angiogram; (5) images with fixation error; or (6) segmentation failure.

The peripapillary region of optic nerve head was divided into eight sectors following the regionalization previously described by Garway-Heath (17), and pVD and pRNFL thickness in each sector was calculated. Including: temporal superior (TS), temporal inferior (TI), superior temporal (ST), inferior temporal (IT), superior nasal (SN), inferior nasal (IN), nasal superior (NS), and nasal inferior (NI).

### Visual field (VF) testing

**Figure 3:**
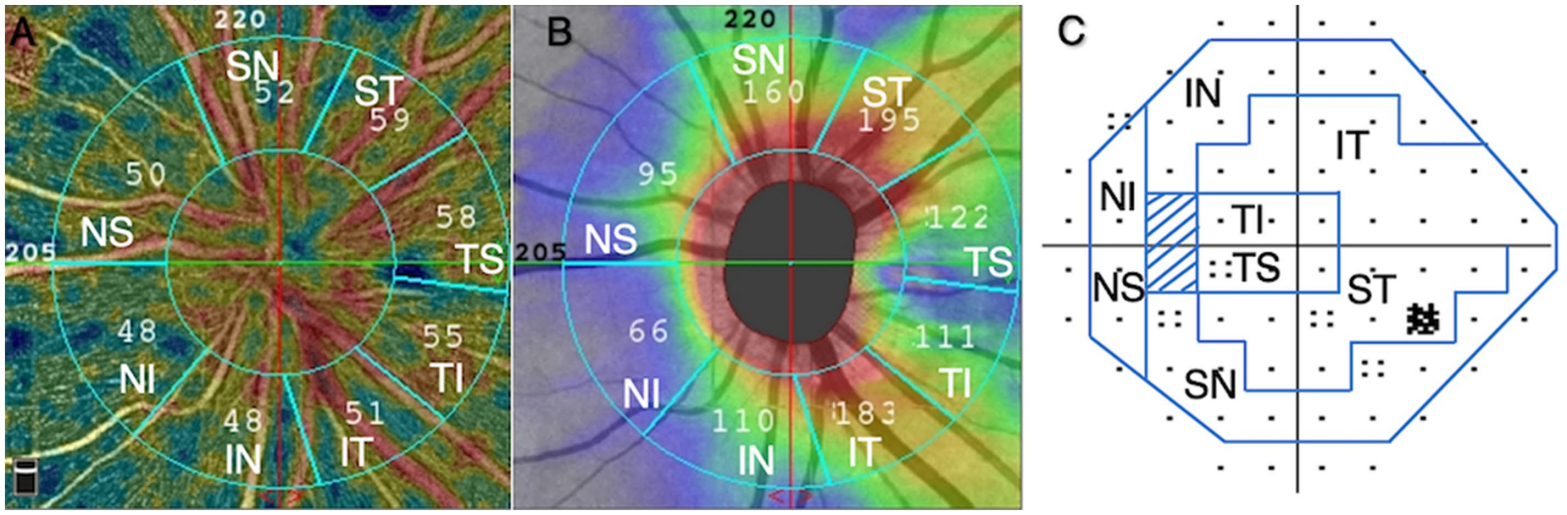
Based on Garway-Heath regionalization, eight regions **(A–C)**. **(A)** peripapillary vessel density, **(B)** peripapillary retinal nerve fiber layer, **(C)** visual field. ST, superior temporal; TS, temporal superior; TI, temporal inferior; IT, inferior temporal; IN, inferior nasal; NI, nasal inferior; NS, nasal superior; SN, superior nasal.

Automated visual field tests were performed with the 30–2 SITA standard program on the Humphrey 750i Visual Field Analyzer (Carl Zeiss Meditec, Dublin, CA, USA) and were grouped into eight sectors that matched the structure (Figure 3) (17). Mean deviation (MD) and pattern standard deviation (PSD) were analyzed. A reliable visual field test was defined: fixation loss rate < 20%, false positive rate < 15%, false negative rate < 15%. The severity of glaucomatous damage was classified as early stage (VF mean deviation (MD) ≥ −6 dB), moderate stage (−12 ≤ MD < −6 dB), and advanced stage (MD < −12 dB).

### Data processing of visual field mean sensitivity (VFMS)

Based on our previous research method (9), the VFMS at various sectors were defined as the average value of the differential light sensitivity (DLS) obtained at VF test locations corresponding to pVD and pRNFL sectors. The VF used logarithmic units of measurement, decibels (dB), measuring the DLS of the subject, and expressed as non-log-transformed 1/L. When directly analyzing the correlation between the dB value and pVD or pRNFL, a logarithmic curve relationship was obtained (18). According to the formula dB = −10 × log10(L), dB values can be divided by 10 to obtain the log10 values, and then calculate the non-logarithmic to get 1/L (1/L represents the maximum brightness of the instrument/the brightness of the visual object actually detected, L: luminance measured in Lamberts) (19). VFMS presents the average value calculated from each area for l/L, according to the eight sectors of pVD or pRNFL. Then the vasculature-function or structure–function relationships were analyzed by comparing the pVD or pRNFL to the corresponding VFMS.

### Propensity score matching

Propensity scores were estimated using a multivariate logistic regression model with glaucoma as a dependent variable and potential confounders of age and AL as covariates. Using a nearest neighbor matching algorithm with a matching ratio 1:1, the caliper value was set at 0.02. A caliper value of 0.02 was considered to be relatively moderate. It can ensure a certain sample size, effectively balance the covariables, and concurrently reduce the differences between groups (20).

### Statistical analysis

All statistical analyses were performed using SPSS software version 26.0 (IBM Corp., Armonk, NY, USA) and R language version 4.2.3. (1) By K-S single sample test, the population was normal distribution, descriptive statistical analyses [mean ± standard deviation (SD) were used in the evaluation of the data; categorical variables were presented as counts and percentages and were analyzed using the chi-square test]. Differences between groups were compared using Student’s t-test, and differences among more than two groups were compared using ANOVA (One-way analysis of variance). (2) The correlation between pVD, pRNFL, and visual function was analyzed using the Pearson correlation coefficient (r). Given that the data demonstrated a normal distribution, we used the Pearson correlation coefficient to evaluate the linear relationship between two continuous variables. (3) Univariate linear regression analyses were performed to examine the associations between pVD, pRNFL, and VFMS. (4) Receiver operating characteristic (ROC) curves were calculated to indicate the separation between glaucoma and control groups. The area under the receiver operating characteristic curves (AUC) was calculated and compared. The Delong method was used to ascertain the statistical significance of AUC differences. An AUC > 0.7 indicated that the classifier provides clinically meaningful discriminative ability. Multiple comparison corrections for ROC analyses were performed using the Bonferroni method, setting a significance threshold of *p* < 0.00625. Corrected *p*-values are available upon request or are presented in the Supplementary material. (5) Statistical significance was set at *p* < 0.05.

## Results

### Demographic and clinical characteristics of patients in four groups

A total of 191 eyes diagnosed with glaucoma and myopia were matched to 580 eyes diagnosed with myopia without glaucoma. Following the matching process (illustrated in Figure 1), 382 eyes were included in the study. These were divided into four groups: 101 eyes with highly myopic glaucoma (HMG, Group 1), 101 eyes with high myopia (HM) without glaucoma (Group 2), 90 eyes with non-highly myopic glaucoma (NHMG, Group 3), and 90 eyes with non-high myopia (NHM) without glaucoma (Group 4). The demographic and clinical characteristics of the four groups were summarized in Table 1. Prior to matching, there was a significant difference in baseline age and axial length (AL) between the glaucoma patients and their corresponding myopia control groups (*p* < 0.05). The standardized differences of age and AL was greater than 20%. After propensity score matching, no significant differences were observed in age and AL parameters between the two paired groups (*p* > 0.05). The standardized differences of age and AL were controlled within 20%. Through Propensity Score Matching (PSM), the balance of various covariates has been significantly improved.

**Table 1:** Baseline characteristics of myopic glaucoma patients before and after propensity score matching.

Parameters	Before matching				After matching			
	Glaucoma	Control	Standardized difference %	*p*	Glaucoma	Control	Standardized difference %	p*
High myopia (*n* = 101:393:101)								
Sex (Male: Female)	65:36	126:267	32.4	<0.001	65:36	44:57	20.8	0.003
Age, y	43.53 (15.56)	32.05 (10.11)	100.5	<0.001	43.53 (15.56)	44.30 (12.60)	−5.4	0.702
Spherical equivalent, D	−9.02 (2.30)	−7.93 (3.10)	−36.9	0.001	−9.02 (2.30)	−9.83 (2.43)	−34.2	0.016
BCVA, log MAR	0.23 (0.41)	0.02 (0.09)	104.5	<0.001	0.23 (0.41)	0.05 (0.14)	64.1	<0.001
Intraocular pressure, mmHg	17.45 (4.65)	16.09 (2.40)	45.1	0.005	17.45 (4.65)	16.10 (2.55)	36.7	0.012
Central corneal thickness, μm	537.15 (38.49)	520.15 (30.89)	52.2	<0.001	537.15 (38.49)	531.10 (32.42)	17.1	0.229
Axial length, mm	27.24 (1.28)	26.48 (1.43)	54.2	<0.001	27.24 (1.28)	27.42 (1.26)	−14.2	0.325
Mean deviation, dB	−11.34 (8.77)	−2.62 (1.76)	−205	<0.001	−11.34 (8.77)	−3.14 (1.72)	−130	<0.001
Pattern standard deviation, dB	8.54 (4.27)	2.57 (1.31)	265.1	<0.001	8.54 (4.27)	3.27 (1.61)	163.3	<0.001
Non-high myopia (*n* = 90:187:90)								
Sex (Male: Female)	51:39	73:114	17.8	0.006	51:39	29:61	24.5	0.001
Age, y	51.82 (14.48)	30.14 (11.16)	171.8	<0.001	51.82 (14.48)	49.84 (9.13)	16.4	0.275
Spherical equivalent, D	−3.21 (1.71)	−4.04 (1.66)	49.6	<0.001	−3.21 (1.71)	−3.71 (1.61)	30.2	0.043
BCVA, log MAR	0.20 (0.30)	−0.01 (0.05)	118.6	<0.001	0.20 (0.30)	0.03 (0.09)	82.5	<0.001
Intraocular pressure, mmHg	19.06 (9.20)	15.62 (2.74)	57.8	0.001	19.06 (9.20)	15.52 (2.51)	58.5	0.001
Central corneal thickness, μm	529.38 (31.30)	526.44 (34.89)	8.9	0.481	529.38 (31.30)	535.10 (35.47)	−17.1	0.253
Axial length, mm	24.81 (1.69)	25.17 (1.14)	−26.7	0.073	24.81 (1.69)	24.67 (1.03)	10.2	0.482
Mean deviation, dB	−10.57 (8.43)	−1.90 (1.35)	−149	<0.001	−10.57 (8.43)	−1.74 (2.04)	−149.2	<0.001
Pattern standard deviation, dB	7.57 (4.15)	2.16 (0.92)	196.2	<0.001	7.57 (4.15)	2.33 (1.45)	168.5	<0.001

BCVA, best-corrected visual acuity. *p*, Pairwise comparison results; p*, Comparison between glaucoma and propensity score matching control group. When the absolute value of the standardized difference is less than 0.2 (20%), the balance of covariates between groups is considered to be satisfactory.

### Results of pVD, pRNFL, and VFMS assessments

**Figure 4:**
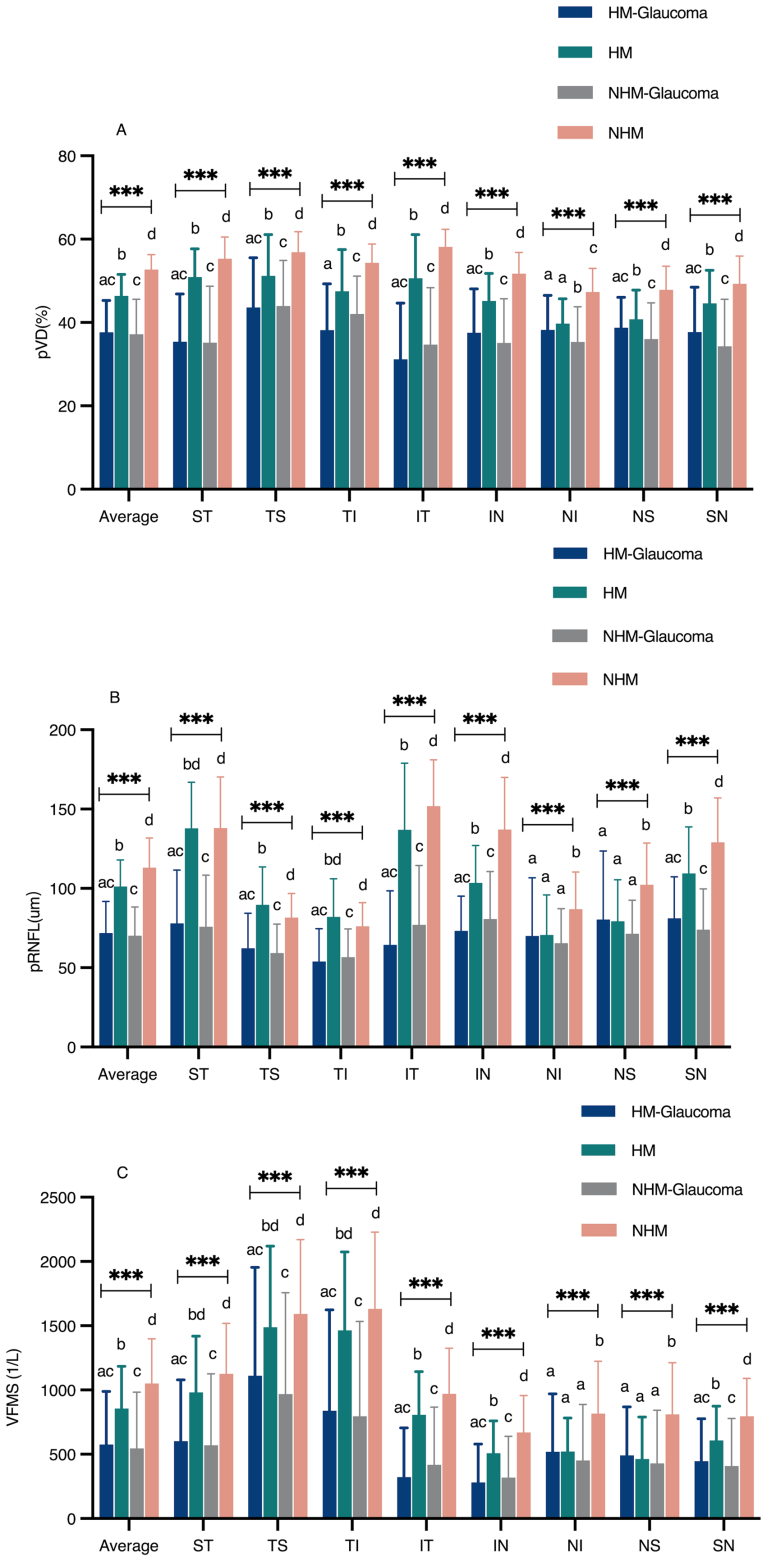
The comparison of peripapillary vessel density (pVD), peripapillary retinal nerve fiber layer (pRNFL), and visual field mean sensitivity (VFMS) among the four groups **(A–C)**. a, b, c, Statistical differences between groups were indicated by letters, with groups sharing at least one similar letter showing no statistically significant difference (*p* ≥ 0.05), and groups with all different letters indicating a significant difference (*p < 0.05, ** *p* < 0.01, *** *p* < 0.001). ST, superior temporal; TS, temporal superior; TI, temporal inferior; IT, inferior temporal; IN, inferior nasal; NI, nasal inferior; NS, nasal superior; SN, superior nasal.

The comparisons of average and regional pVD, pRNFL and VFMS are summarized in Table 2 and Figure 4. In the analysis of HMG eyes (Group 1) versus HM without glaucoma eyes (Group 2), the average pVD was significantly lower in Group 1 (37.66 ± 7.61) compared to Group 2 (46.40 ± 5.16, *p* < 0.001). The pVD in all eight regions was significantly reduced in Group 1 compared to Group 2 (*p* < 0.05), except for the NI region (*p* = 0.147). When comparing NHMG eyes (Group 3) with NHM without glaucoma eyes (Group 4), the average pVD was also significantly lower in Group 3 (37.18 ± 8.43) than in Group 4 (52.69 ± 3.60, *p* < 0.001). The pVD in all eight regions in Group 3 was significantly reduced compared to Group 4 (p < 0.001). Multiple comparison analyses showed no significant differences in average and regional pVD between Group 1 and Group 3 (*p* > 0.05), except for the TI and NI regions (*p* < 0.05). Both glaucoma groups (Group 1 and 3) had significantly lower average and regional pVD compared to their respective control groups (Group 2 and 4) (*p* < 0.05), except for the NI and NS regions (*p* > 0.05). Comparisons between Group 2 and 4 indicated that high myopia led to decreased pVD across all regions (*p* < 0.05).

**Table 2:** Basic characteristics of average and eight regional pVD, pRNFL and VFMS in four groups.

Parameters	Highly myopia			Non-highly myopia			p*	Pair comparison (group)
	Glaucoma (1) (*n* = 101)	Control (2) (*n* = 101)	*p*	Glaucoma (3) (*n* = 90)	Control (4) (*n* = 90)	*p*		
Peripapillary vessel density (pVD), %								
Average	37.66 (7.61)	46.40 (5.16)	<0.001	37.18 (8.43)	52.69 (3.60)	<0.001	<0.001	1 = 3 < 2 < 4
Superior temporal	35.39 (11.47)	50.91 (6.8)	<0.001	35.19 (13.50)	55.33 (5.21)	<0.001	<0.001	1 = 3 < 2 < 4
Temporal superior	43.59 (11.93)	51.19 (9.91)	<0.001	43.97 (10.97)	56.90 (4.89)	<0.001	<0.001	1 = 3 < 2 < 4
Temporal inferior	38.20 (11.07)	47.51 (10.00)	<0.001	42.06 (9.06)	54.36 (4.51)	<0.001	<0.001	1 < 3 < 2 < 4
Inferior temporal	31.17 (13.50)	50.64 (10.44)	<0.001	34.71 (13.65)	58.14 (4.23)	<0.001	<0.001	1 = 3 < 2 < 4
Inferior nasal	37.51 (10.59)	45.2 (6.57)	<0.001	35.11 (10.60)	51.72 (5.07)	<0.001	<0.001	1 = 3 < 2 < 4
Nasal inferior	38.26 (8.27)	39.73 (5.96)	0.147	35.34 (8.46)	47.30 (5.72)	<0.001	<0.001	3 < 1 = 2 < 4
Nasal superior	38.74 (7.31)	40.77 (6.99)	0.045	36.04 (8.66)	47.83 (5.69)	<0.001	<0.001	1 = 3 = 2 < 4
Superior nasal	37.69 (10.76)	44.6 (7.95)	<0.001	34.32 (11.29)	49.27 (6.66)	<0.001	<0.001	1 = 3 < 2 < 4
Peripapillary retinal nerve fiber layer thickness (pRNFL), μm								
Average	71.13 (18.66)	101.22 (16.79)	<0.001	70.14 (18.09)	113.02 (18.83)	<0.001	<0.001	1 = 3 < 2 < 4
Superior temporal	77.96 (33.62)	137.85 (29.01)	<0.001	75.83 (32.48)	138.06 (32.21)	<0.001	<0.001	1 = 3 < 2 = 4
Temporal superior	62.34 (22.07)	89.64 (23.93)	<0.001	59.26 (18.23)	81.66 (15.20)	<0.001	<0.001	1 = 3 < 4 < 2
Temporal inferior	53.97 (20.64)	82.06 (23.99)	<0.001	56.61 (17.78)	76.18 (14.78)	<0.001	<0.001	1 = 3 < 2 = 4
Inferior temporal	64.42 (34.05)	136.91 (41.96)	<0.001	77.09 (37.40)	151.86 (29.24)	<0.001	<0.001	1 = 3 < 2 < 4
Inferior nasal	73.27 (21.90)	103.46 (23.64)	<0.001	80.66 (29.99)	137.18 (32.80)	<0.001	<0.001	1 = 3 < 2 < 4
Nasal inferior	67.55 (22.86)	70.62 (25.31)	0.906	65.42 (21.80)	86.99 (23.31)	<0.001	<0.001	1 = 3 = 2 < 4
Nasal superior	77.29 (26.85)	79.25 (26.3)	0.815	71.37 (21.19)	102.36 (26.17)	<0.001	<0.001	1 = 3 = 2 < 4
Superior nasal	81.12 (26.22)	109.36 (29.42)	<0.001	74.00 (25.65)	129.10 (27.91)	<0.001	<0.001	1 = 3 < 2 < 4
Visual field mean sensitivity (VFMS), 1/L								
Average	576.38 (412.96)	855.11 (328.49)	<0.001	545.01 (439.04)	1051.31 (347.61)	<0.001	<0.001	1 = 3 < 2 < 4
Superior temporal	601.54 (478.34)	982.27 (437.22)	<0.001	570.44 (555.44)	1126.75 (391.45)	<0.001	<0.001	1 = 3 < 2 = 4
Temporal superior	1110.54 (843.54)	1489.01 (630.27)	<0.001	968.16 (789.23)	1590.99 (578.47)	<0.001	<0.001	1 = 3 < 2 = 4
Temporal inferior	839.00 (785.56)	1465.02 (608.72)	<0.001	795.07 (738.87)	1631.14 (597.93)	<0.001	<0.001	1 = 3 < 2 = 4
Inferior temporal	322.90 (382.19)	805.94 (336.72)	<0.001	417.26 (449.13)	970.9 (354.93)	<0.001	<0.001	1 = 3 < 2 < 4
Inferior nasal	280.64 (298.51)	507.60 (252.66)	<0.001	318.07 (322.36)	668.69 (288.31)	<0.001	<0.001	1 = 3 < 2 < 4
Nasal inferior	518.57 (452.17)	521.17 (260.96)	0.960	452.63 (435.17)	815.98 (407.97)	<0.001	<0.001	1 = 3 = 2 < 4
Nasal superior	492.10 (376.50)	462.18 (326.71)	0.547	429.85 (412.06)	811.4 (401.11)	<0.001	<0.001	1 = 3 = 2 < 4
Superior nasal	445.77 (330.89)	607.70 (267.16)	<0.001	408.62 (369.24)	794.63 (294.98)	<0.001	<0.001	1 = 3 < 2 < 4

*p*, Glaucoma and myopia comparison results. p*, Multiple comparison results.

Group 1 showed significantly thinner average pRNFL thickness (71.13 ± 18.66 μm) compared to Group 2 (101.22 ± 16.79 μm, *p* < 0.001), with reductions in all regions (*p* < 0.001) except NI (*p* = 0.906) and NS (*p* = 0.815). Similar reductions were observed between Groups 3 and 4 (70.14 ± 18.09 μm vs. 113.02 ± 18.83 μm, *p* < 0.001). No significant differences in pRNFL were found between Groups 1 and 3 (*p* > 0.05). Both glaucoma groups (Groups 1 and 3) had thinner average and regional pRNFL than controls (Groups 2 and 4) (*p* < 0.05), except in NI and NS regions (*p* > 0.05). High myopia was associated with reduced average and regional pRNFL thickness (*p* < 0.05).

Average VFMS was significantly lower in Group 1 (576.38 ± 412.96) compared to Group 2 (855.11 ± 328.49, *p* < 0.001), with similar regional reductions (*p* < 0.001), except in NI and NS regions (*p* > 0.05). Comparable trends were observed between Groups 3 and 4 (545.01 ± 439.04 vs. 1051.31 ± 347.61, *p* < 0.001). No significant differences in VFMS were found between Groups 1 and 3 (*p* > 0.05). Both glaucoma groups (Groups 1 and 3) showed lower VFMS compared to controls (Groups 2 and 4) (*p* < 0.05), excluding NI and NS regions (*p* > 0.05).

### Correlations between structure (pVD, pRNFL) and function (VFMS)

**Figure 5:**
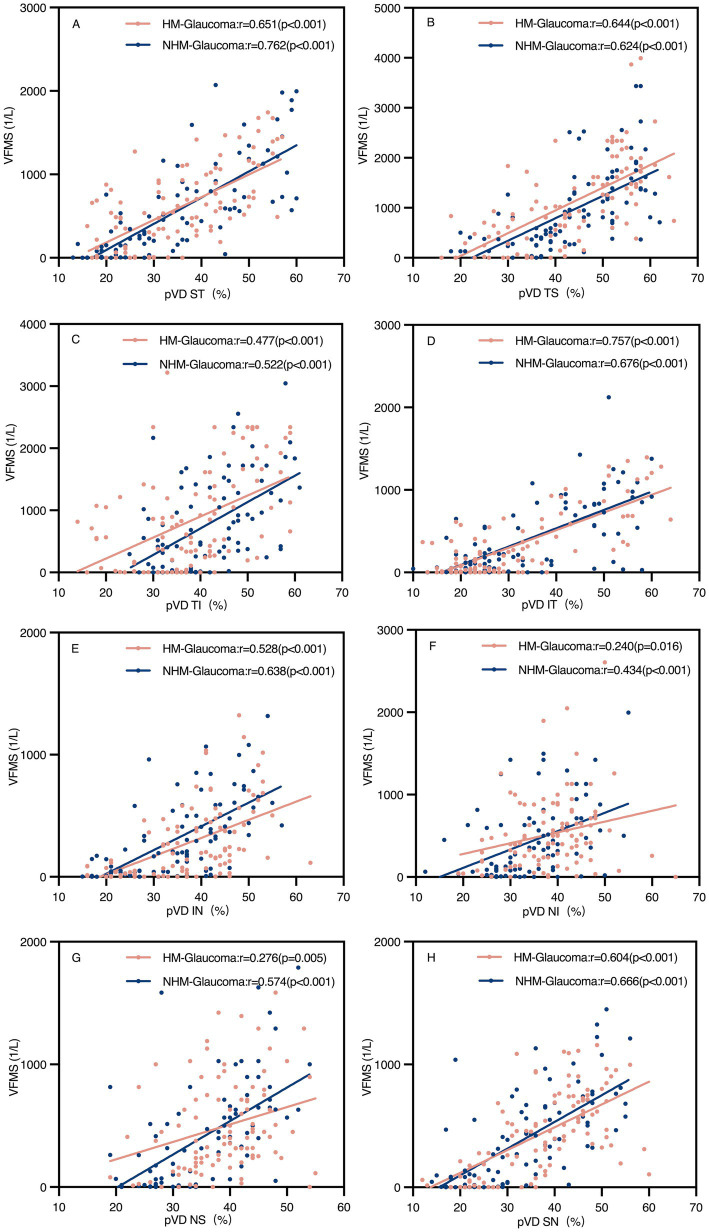
Scatter plots of peripapillary vessel density (pVD) and visual field mean sensitivity (VFMS) in glaucoma groups **(A–H)**. The pVD-VFMS correlation in all regions was significant in both glaucoma groups (*p* < 0.05). r, Pearson correlation coefficient; HM, high myopia; NHM, non-high myopia; ST, superior temporal; TS, temporal superior; TI, temporal inferior; IT, inferior temporal; IN, inferior nasal; NI, nasal inferior; NS, nasal superior; SN, superior nasal.

Table 3 summarizes the correlation analyses between pVD, pRNFL, and VFMS in Group 1 and Group 3. Significant positive correlations were found between average pVD and average VFMS in both groups (*r* = 0.681 and *r* = 0.749, respectively, *p* < 0.001). Similarly, average pRNFL correlated with average VFMS in both groups (*r* = 0.504 and *r* = 0.722, respectively, *p* < 0.001). In both groups, the correlation between average pVD and average VFMS was stronger than that between average pRNFL and average VFMS. as clearly depicted in Figure 5, which presents a scatter plot visualization of these correlations.

**Table 3:** The correlation analyses of structure–function in the average and eight regions.

Regions	Highly myopic glaucoma (*n* = 101)				Non-highly myopic glaucoma (*n* = 90)			
	pVD-VFMS		pRNFL-VFMS		pVD-VFMS		pRNFL-VFMS	
	*r*	*p*	*r*	*p*	*r*	*p*	*r*	*p*
Average	0.681	<0.001	0.504	<0.001	0.749	<0.001	0.722	<0.001
Superior temporal	0.651	<0.001	0.668	<0.001	0.762	<0.001	0.782	<0.001
Temporal superior	0.644	<0.001	0.584	<0.001	0.624	<0.001	0.484	<0.001
Temporal inferior	0.477	<0.001	0.475	<0.001	0.522	<0.001	0.444	<0.001
Inferior temporal	0.757	<0.001	0.833	<0.001	0.676	<0.001	0.721	<0.001
Inferior nasal	0.528	<0.001	0.648	<0.001	0.638	<0.001	0.482	<0.001
Nasal inferior	0.240	0.016	0.006	0.955	0.434	<0.001	0.172	0.106
Nasal superior	0.276	0.005	−0.124	0.215	0.574	<0.001	0.439	<0.001
Superior nasal	0.604	<0.001	0.488	<0.001	0.666	<0.001	0.704	<0.001

pVD, peripapillary vessel density; pRNFL, peripapillary retinal nerve fiber layer; VFMS, visual field mean sensitivity. r, Pearson correlation coefficient.

**Figure 6:**
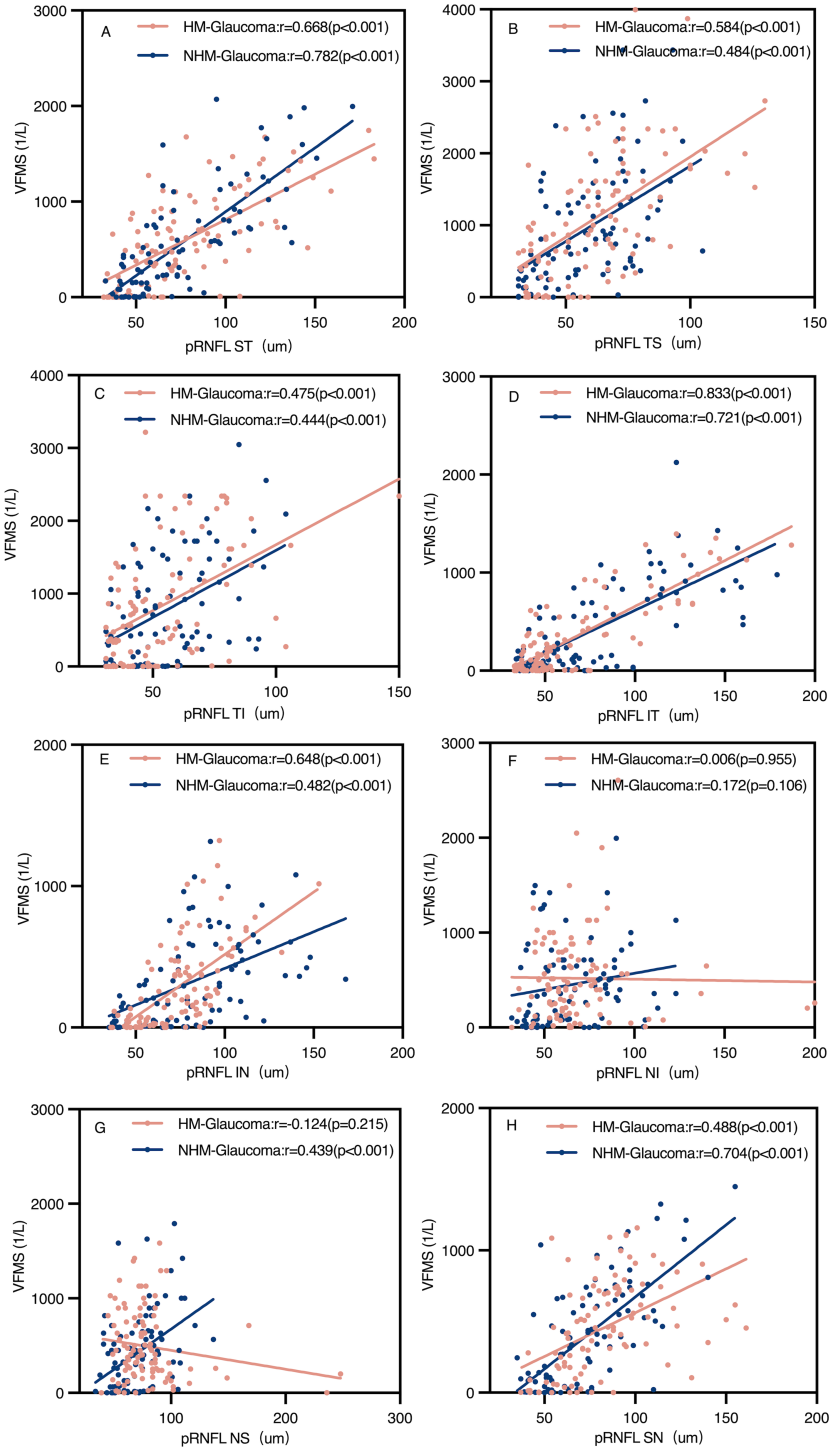
Scatter plots of peripapillary retinal nerve fibre layer (pRNFL) and visual field mean sensitivity (VFMS) in glaucoma groups **(A–H)**. The pRNFL-VFMS correlation in all regions was significant in both glaucoma groups (*p* < 0.05), except the NI region and NS region of pRNFL in the highly myopic glaucoma group and NI region of pRNFL in the non-highly myopic glaucoma group. r, Pearson correlation coefficient; HM, high myopia; NHM, non-high myopia; ST, superior temporal; TS, temporal superior; TI, temporal inferior; IT, inferior temporal; IN, inferior nasal; NI, nasal inferior; NS, nasal superior; SN, superior nasal.

Regional analyses revealed significant positive correlations between pVD and VFMS across all regions in both groups (*p* < 0.05). However, pRNFL and VFMS no significant correlations were observed in NI and NS regions in Group 1 and NI region in Group 3. The strongest correlation between pVD-VFMS and pRNFL-VFMS was in the ST and IT regions for both groups. Notably, the pRNFL-VFMS correlation was stronger than the pVD-VFMS correlation in ST and IT regions (*p* < 0.001). Figure 6 further complements these findings by illustrating the regional correlation patterns, offering a detailed spatial perspective on the relationships.

Further analysis of pVD, pRNFL, and MD by visual field loss severity is presented in Table 4. In early-stage glaucoma with high myopia, pVD was positively correlated with MD (*p* < 0.05), while pRNFL had no significant correlation with MD. In moderate-to-advanced glaucoma, both pVD and pRNFL showed significant correlations with MD. In non-highly myopic glaucoma, both pVD and pRNFL were positively correlated with MD in early and moderate-to-advanced stages (*p* < 0.05).

**Table 4:** Correlation analysis of the structure and function in early and moderate-advanced stage glaucoma.

Stages	Highly myopic glaucoma				Non-highly myopic glaucoma			
	pVD-MD		pRNFL-MD		pVD-MD		pRNFL-MD	
	*r*	*p*	*r*	*p*	*r*	*p*	*r*	*p*
Early stage	0.436	0.010	0.153	0.387	0.495	0.003	0.523	0.001
Moderate-advanced stage	0.558	<0.001	0.539	<0.001	0.453	0.001	0.343	0.010

pVD, peripapillary vessel density; pRNFL, peripapillary retinal nerve fiber layer; MD, mean deviation.

### Linear regression analysis

**Figure 7:**
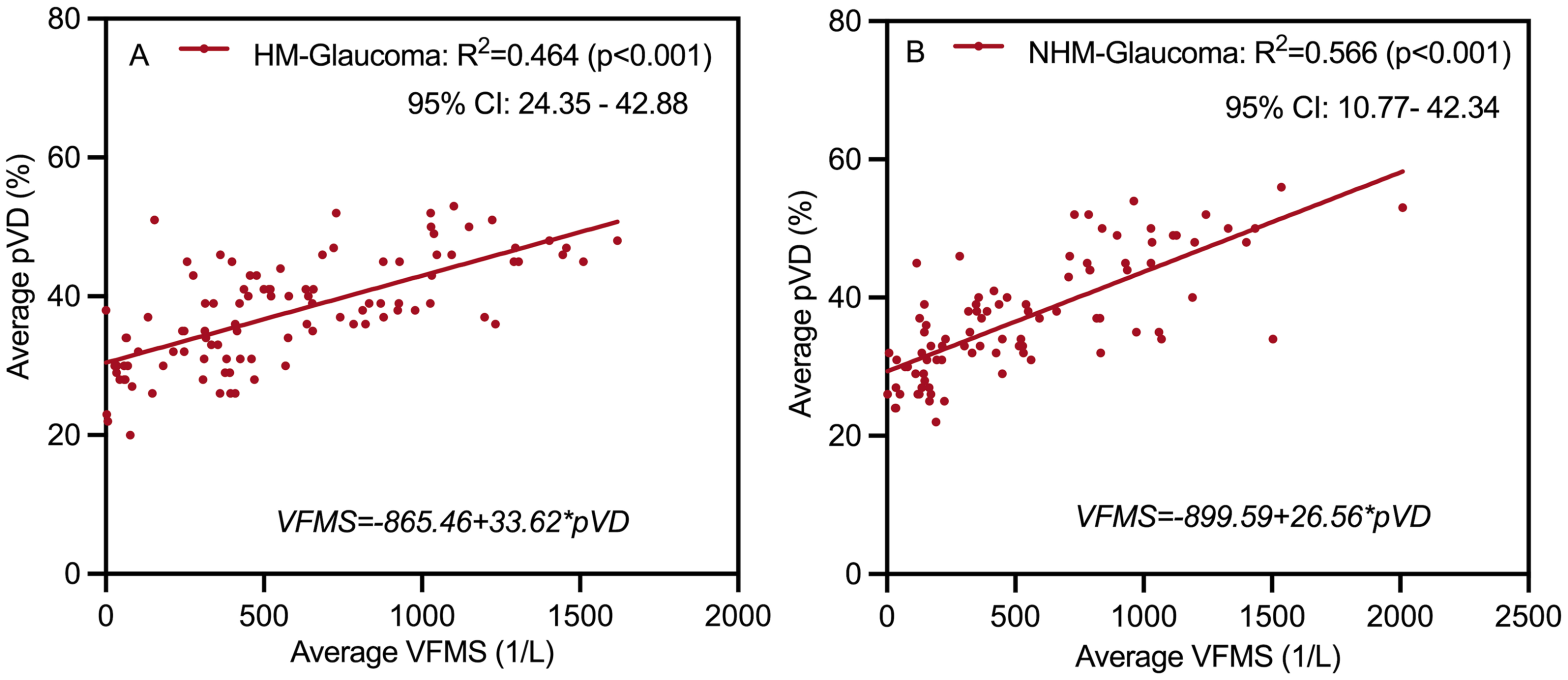
Multivariate linear regression analysis scatter plot to identify structure associated with function at **(A,B)**. Peripapillary vessel density (pVD) significantly affects visual field mean sensitivity (VFMS) in highly myopic glaucoma and non-highly myopic glaucoma group. R^2^, Coefficient of determination; HM, high myopia; NHM, non-high myopia.

Linear regression analyses were further conducted between average pVD, pRNFL, and VFMS (Table 5, Figure 7). The results of univariate linear regression analysis indicated that pVD had a significant impact on VFMS in both HMG group (*R^2^* = 0.459, *p* < 0.001) and NHMG group (*R^2^* = 0.556, *p* < 0.001). Similarly, pRNFL also showed a significant effect on VFMS in two groups. In the multivariate linear regression analysis, pVD maintained a significant correlation with VFMS in HMG group (*R^2^* = 0.464, *p* < 0.001) and the NHMG group (*R^2^* = 0.566, *p* = 0.001). However, we observed that pRNFL did not significantly impact VFMS in either group (*p* = 0.172; *p* = 0.082, respectively).

**Table 5:** Univariate and multivariate linear regression analysis of the structure and function.

Parameters	Univariate			Multivariate			
	Regression coefficient (95% CI)	*p*	*R^2^*	Regression coefficient (95% CI)	*p*	*R^2^*	*VIF*
Highly myopic glaucoma (*n* = 101)							
pVD	36.98 (29.06–44.90)	<0.001	0.459	33.62 (24.35–42.88)	<0.001	0.464	1.379
pRNFL	9.17 (5.47–12.87)	<0.001	0.188	2.477 (−1.09–5.98)	0.172		
Non-highly myopic glaucoma (*n* = 90)							
pVD	38.99 (31.69–46.30)	<0.001	0.556	26.56 (10.77–42.34)	0.001	0.566	4.775
pRNFL	17.53 (13.97–21.08)	<0.001	0.516	6.52 (−0.84–13.88)	0.082		

pVD, peripapillary vessel density; pRNFL, peripapillary retinal nerve fiber layer; VIF, variance inflation factor. *R*^2^, Coefficient of determination.

### ROC curve analysis

Table 6 summarizes the ROC curve analysis assessing the diagnostic capabilities of pVD and pRNFL in HMG and NHMG. Both average and eight regional pVD measurements showed diagnostic significance in both groups, except for the NI region in Group 1. Average pVD had significantly higher diagnostic ability in Group 3 (AUC: 0.945) compared to Group 1 (AUC: 0.822; p < 0.001). In Group 1, the best diagnostic performance for pVD was at the average level, ST and IT region. In Group 3, the most effective diagnostic regions were the average level, ST, IT, and IN regions. Combining the ST and IT regions improved diagnostic accuracy for both groups. Additionally, pVD demonstrated greater diagnostic capability in NHMG than in HMG (*p* = 0.016).

**Table 6:** Receiver operating characteristic curve analysis of the diagnostic ability of pVD and pRNFL for glaucoma.

Regions	Highly myopic glaucoma		Non-highly myopic glaucoma		p*
	AUC (95%CI)	*p*	AUC (95%CI)	*p*	
Peripapillary vessel density (pVD) (%)					
Average	0.822 (0.764–0.879)	<0.001	0.945 (0.912–0.977)	<0.001	<0.001
Superior temporal	0.865 (0.814–0.916)	<0.001	0.894 (0.843–0.944)	<0.001	0.436
Temporal superior	0.697 (0.625–0.769)	<0.001	0.859 (0.805–0.913)	<0.001	<0.001
Temporal inferior	0.748 (0.678–0.817)	<0.001	0.874 (0.818–0.929)	<0.001	0.006
Inferior temporal	0.850 (0.795–0.905)	<0.001	0.949 (0.918–0.979)	<0.001	0.002
Inferior nasal	0.719 (0.649–0.79)	<0.001	0.923 (0.884–0.961)	<0.001	<0.001
Nasal inferior	0.555 (0.475–0.635)	0.176	0.876 (0.826–0.927)	<0.001	<0.001
Nasal superior	0.591 (0.513–0.67)	0.025	0.866 (0.815–0.917)	<0.001	<0.001
Superior nasal	0.701 (0.629–0.773)	<0.001	0.875 (0.821–0.93)	<0.001	<0.001
Combine ST + IT	0.905 (0.863–0.947)	<0.001	0.965 (0.941–0.989)	<0.001	0.016
Peripapillary retinal nerve fiber layer (pRNFL) (μm)					
Average	0.881 (0.832–0.930)	<0.001	0.948 (0.918–0.978)	<0.001	0.023
Superior temporal	0.906 (0.863–0.948)	<0.001	0.903 (0.858–0.948)	<0.001	0.930
Temporal superior	0.819 (0.760–0.879)	<0.001	0.824 (0.764–0.884)	<0.001	0.914
Temporal inferior	0.827 (0.770–0.884)	<0.001	0.803 (0.738–0.867)	<0.001	0.581
Inferior temporal	0.907 (0.867–0.948)	<0.001	0.923 (0.884–0.963)	<0.001	0.584
Inferior nasal	0.826 (0.770–0.882)	<0.001	0.900 (0.855–0.944)	<0.001	0.043
Nasal inferior	0.535 (0.455–0.615)	0.387	0.753 (0.683–0.823)	<0.001	<0.001
Nasal superior	0.510 (0.429–0.590)	0.814	0.822 (0.762–0.882)	<0.001	<0.001
Superior nasal	0.775 (0.711–0.840)	<0.001	0.923 (0.886–0.961)	<0.001	<0.001
Combine ST + IT	0.934 (0.898–0.970)	<0.001	0.942 (0.909–0.975)	<0.001	0.750

AUC, area under the curve. p*, Comparison of diagnostic ability between highly myopic glaucoma and non-highly myopic glaucoma group, the statistical significance between AUC curves were calculated based on DeLong methods.

**Figure 8:**
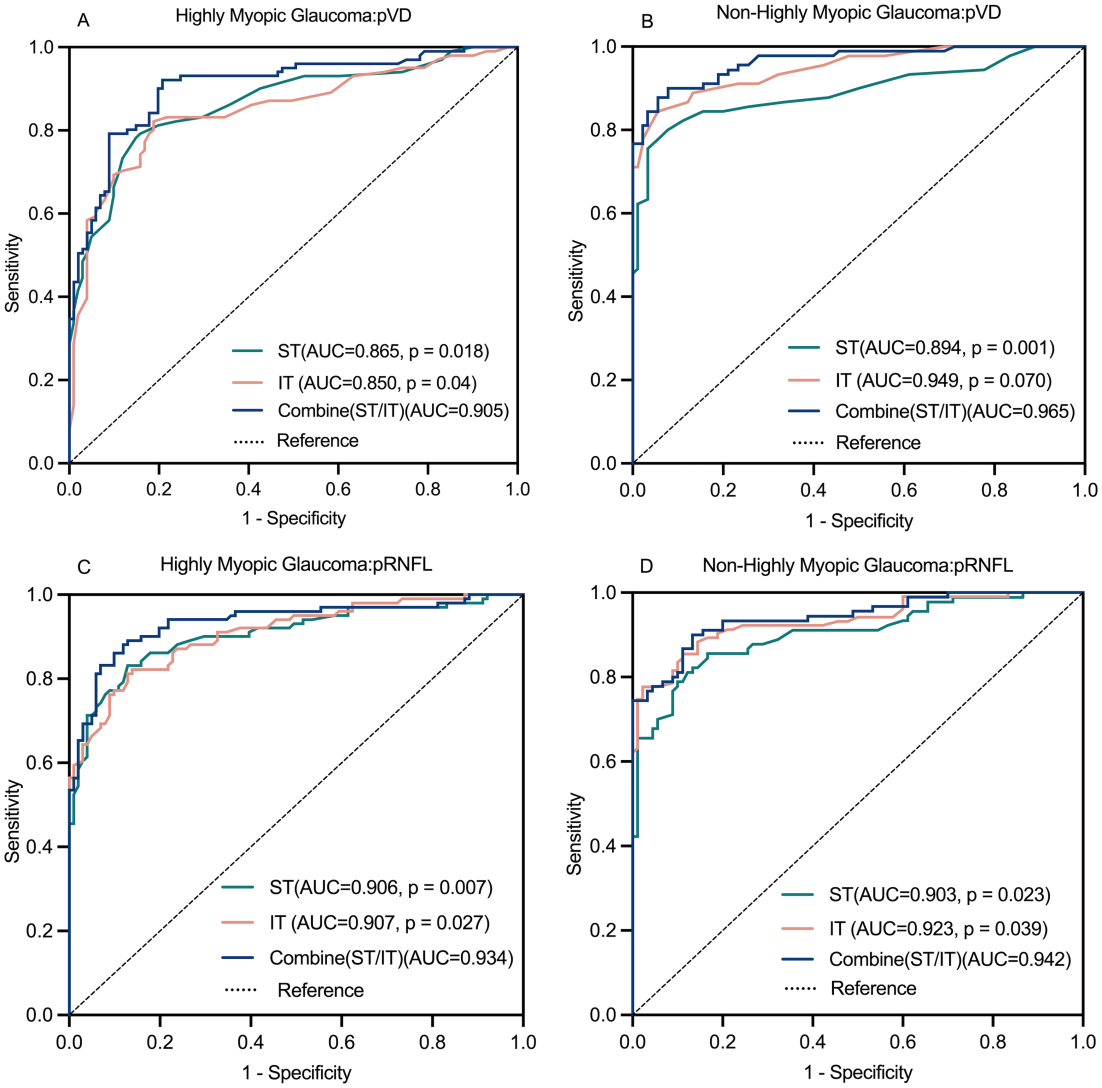
Receiver operating characteristics (ROC) curve for pVD and pRNFL diagnostic ability **(A–D)**. The combined diagnostic ability of pVD and pRNFL was superior to the individual diagnostic capabilities of the ST region and IT region. pVD, peripapillary vessel density; pRNFL, peripapillary retinal nerve fiber layer; ST, superior temporal; IT, inferior temporal.

The ROC analysis also revealed strong diagnostic abilities for average and regional pRNFL measurements in both groups, except for the NI and NS regions in Group 1. Average pRNFL had significantly higher diagnostic efficacy in Group 3 (AUC: 0.948) compared to Group 1 (AUC: 0.881; *p* = 0.023). In Group 1, the top diagnostic markers for pRNFL were the average level, ST, and IT region. In Group 3, the most robust performance was seen in the average level, ST, IT, and SN region. Combining the ST and IT regions improved diagnostic performance in both groups, with no significant differences between the two groups for this combination (*p* = 0.750). Figure 8 illustrates these findings.

### Comparing the diagnostic capabilities of pVD and pRNFL

Table 7 compares the diagnostic performance of pVD and pRNFL for glaucoma in highly myopic and non-highly myopic eyes. In HMG, average pRNFL demonstrated significantly better diagnostic performance (AUC: 0.881) than average pVD (AUC: 0.822, *p* = 0.026). Similarly, when combining the ST and IT regions, pRNFL showed superior diagnostic ability compared to pVD (AUC: 0.934 vs. 0.905, *p* = 0.010). In NHMG, no significant differences were observed between the diagnostic capabilities of average pVD and pRNFL (AUC: 0.945 vs. 0.948, *p* = 0.763) or the combined ST and IT regions (AUC: 0.965 vs. 0.942, *p* = 0.105).

**Table 7:** Comparison of diagnostic ability between pVD and pRNFL in myopic glaucoma groups.

Stages	pVD	pRNFL	*p*
	AUC (95%CI)	AUC (95%CI)	
Highly Myopic Glaucoma			
Overall Average	0.822 (0.764–0.879)	0.881 (0.832–0.930)	0.026
Overall Combine ST + IT	0.905 (0.863–0.947)	0.934 (0.898–0.970)	0.010
Early-Average	0.773 (0.660–0.887)	0.809 (0.704–0.913)	0.502
Early-Combine ST + IT	0.850 (0.753–0.947)	0.865 (0.774–0.956)	0.521
Moderate-Average	0.839 (0.762–0.916)	0.859 (0.769–0.949)	0.689
Moderate-Combine ST + IT	0.942 (0.905–0.979)	0.973 (0.949–0.996)	0.034
Advanced-Average	0.962 (0.926–0.998)	0.998 (0.994–1.002)	0.043
Advanced-Combine ST + IT	0.994 (0.984–1.004)	0.998 (0.994–1.002)	0.415
Non-Highly Myopic Glaucoma			
Overall Average	0.945 (0.912–0.977)	0.948 (0.918–0.978)	0.763
Overall Combine ST + IT	0.965 (0.941–0.989)	0.942 (0.909–0.975)	0.105
Early-Average	0.858 (0.775–0.941)	0.906 (0.839–0.972)	0.204
Early-Combine ST + IT	0.895 (0.824–0.965)	0.861 (0.778–0.944)	0.426
Moderate-Average	0.993 (0.976–1.003)	1	0.369
Moderate-Combine ST + IT	1	1	1
Advanced-Average	0.998 (0.993–1.003)	0.997 (0.992–1.003)	0.620
Advanced-Combine ST + IT	1	1	1

pVD, peripapillary vessel density; pRNFL, peripapillary retinal nerve fiber layer; AUC, area under the curve; ST, superior temporal; IT, inferior temporal. Comparison of diagnostic ability by DeLong’s test.

Evaluate the staging diagnostic abilities of pVD and pRNFL in Table 7. In early-stage HMG, no significant differences were noted between the diagnostic abilities of average pVD and pRNFL or their combined ST and IT regions. For moderate-stage HMG, average diagnostic abilities of pVD and pRNFL were comparable, but pRNFL outperformed pVD in the combined ST and IT regions (*p* = 0.034). In advanced-stage HMG, pRNFL showed superior average diagnostic performance (*p* = 0.043), while no significant differences were observed in the combined ST and IT regions.

For NHMG, no significant differences were found in early-, moderate-, or advanced-stage groups between pVD and pRNFL, either at the average level or in the combined ST and IT regions. Across all stages, pVD and pRNFL demonstrated similar diagnostic capabilities in NHMG.

## Discussion

Given the significant influence of aging and axial elongation on parameters such as pRNFL, pVD, and visual function (21–23), our study employed propensity score matching to balance covariate distributions between the glaucoma and control groups, thereby minimizing confounding factors and enhancing the reliability of the findings. We matched participants based on age and AL, while SE was not included in the model due to its high collinearity with AL. The present study demonstrated a consistent downward trend in pVD, pRNFL, and VFMS from the non-high myopia group to the high myopia group, and further to the myopia with glaucoma groups. Visual function loss associated with high myopia was primarily concentrated in the nasal regions of the optic disc, while glaucoma-induced damage predominantly affected the temporal regions. Both pVD and pRNFL were identified as sensitive biomarkers for diagnosing highly myopic glaucoma. While pRNFL showed slightly higher diagnostic accuracy, both biomarkers exhibited comparable diagnostic utility in early-stage glaucoma. Notably, in the early stage of highly myopic glaucoma, pVD demonstrated a stronger correlation with visual function compared to pRNFL, suggesting its potential as a complementary marker for both the diagnosis and functional monitoring of early glaucomatous changes in highly myopic eyes.

### RNFL and pVD: diagnostic patterns and utility

RNFL thinning is a well-established marker of glaucomatous progression, with this study demonstrating progressive reductions from non-high myopic to highly myopic glaucoma. However, structural changes, such as optic disc tilting in high myopia, can alter RNFL distribution, particularly in temporal-superior regions, complicating its use as a reliable biomarker. In contrast, pVD appears to be a more stable and reliable metric in highly myopic patients, as it is less affected by structural changes induced by myopia (19). Previous studies have shown that glaucoma significantly reduces pVD and, with reductions strongly correlating with visual field deterioration, particularly in the TS and TI regions (24). Myopia is also associated with reductions in both pVD and pRNFL (25). In our previous study, pVD reduction was more strongly correlated with high myopia-induced visual function impairment than pRNFL reduction (9). These findings suggest that integrating both structural (pRNFL) and vascular (pVD) metrics may enhance diagnostic precision. Notably, the NI and NS regions showed non-significant differences in pVD and pRNFL between glaucoma and control groups. This may be explained by the fact that nasal sectors are generally less affected in early glaucoma, where damage predominantly occurs in the superior and inferior temporal regions. Furthermore, these nasal regions are more prone to segmentation errors and measurement variability, especially in highly myopic eyes with peripapillary atrophy, potentially reducing the sensitivity of these parameters in those areas (26, 27).

To observe the performance of pVD, pRNFL, and VFMS under varying degrees of myopia in the presence or absence of glaucoma, we conducted ANOVA and *post hoc* multiple comparisons among the four groups. This allowed us to evaluate whether the damage from glaucoma masked or amplified the damage associated with high myopia. Our analysis confirmed a stepwise reduction in pVD across non-high myopia, high myopia, and myopia with glaucoma (Table 2, Figure 4). High myopia caused diffuse and uniform pVD reduction, whereas glaucoma led to more severe, region-specific pVD damage, particularly in the ST and IT regions. Suwan et al. (25) reported more severe pVD damage in myopic glaucoma compared to non-myopic glaucoma, while our study found no significant differences between these groups except in the NI region. However, their subsequent research demonstrated that the extent of pVD damage was comparable between the groups, which aligns with our findings (28). These discrepancies across studies may be attributed to advancements in research methodology, differences in sample selection, or variability in glaucoma severity among study populations.

### Visual function: distinguishing myopia from glaucoma

We observed a significant decline in VFMS in high myopic eyes compared to non-highly myopic eyes. However, this decline was much less severe than in glaucoma groups. High myopia-induced visual function loss was primarily localized to the nasal regions of the optic disc and visual field (NI, NS, IN, and SN regions), while glaucoma-induced damage predominantly affected the temporal regions (ST, TS, TI, and IT regions). These regional differences provided crucial clues for differentiating high myopia from glaucoma. Notably, no significant differences in VFMS or regional analyses were observed between high myopia with glaucoma and non-high myopia with glaucoma groups. We hypothesize that the damage caused by glaucoma may overshadow myopia-induced damage, masking some differences between the two groups.

### Relationships between pVD, pRNFL and VFMS

Our findings demonstrated a significant correlation between pVD and pRNFL thickness with VFMS in both glaucoma groups. Notably, the relationship between pVD and VFMS was stronger than that between pRNFL and VFMS in highly myopic glaucoma and non-highly myopic glaucoma. Across all regions, pVD exhibited a significant positive correlation with VFMS, whereas pRNFL showed no significant association in the NI and NS sectors. Consequently, pVD emerged as a more robust indicator of VFMS than pRNFL. In the comparison across different stages of glaucoma, it can also be observed that the reduction in pVD is significantly positively correlated with the degree of VF damage, while in the early stage of glaucoma in high myopia, there is no correlation between RNFL and VF damage. Linear regression analysis confirmed that pVD was more consistent than pRNFL in monitoring visual function, particularly in the ST and IT regions, which are critical for glaucoma diagnosis. These results suggest that pVD may be a more reliable marker for tracking glaucomatous visual field damage.

### Diagnostic capabilities of pRNFL and pVD in myopic glaucoma

This study evaluated the diagnostic efficacy of pRNFL and pVD across eight peripapillary regions in highly myopic and non-highly myopic glaucoma patients. In highly myopic glaucoma, all regional pRNFL measurements, except for the NI and NS regions, exhibited significant diagnostic capacity, with the ST and IT regions showing the highest performance. For non-highly myopic glaucoma, pRNFL demonstrated strong diagnostic efficiency across all regions, with superior performance in the ST and IT sectors. Our findings revealed that pRNFL exhibited excellent diagnostic performance for glaucoma (AUC: 0.934, 0.942). However, this was slightly lower than the diagnostic ability reported by Fukai et al. (29) (AUC: 0.97), which may be attributed to the inclusion of only myopic glaucoma patients in our study, in contrast to the broader glaucoma population assessed by Fukai et al. (29).

ROC curve analyses of the combined ST and IT regions revealed superior diagnostic accuracy compared to overall or individual regions in both glaucoma groups. Diagnostic evaluation of pVD highlighted its sensitivity for detecting myopic glaucoma. In highly myopic glaucoma, pVD demonstrated strong diagnostic performance in all regions except the NI region, whereas in non-highly myopic glaucoma, pVD performed excellently across all regions, particularly in the ST and IT sectors. Combining the ST and IT regions further enhanced diagnostic efficacy, introducing a novel approach to improving diagnostic accuracy.

Stage-specific analyses revealed that in early and moderate stages of highly myopic glaucoma, pVD and pRNFL exhibited comparable diagnostic abilities. However, in advanced stages, pVD’s diagnostic performance declined significantly compared to pRNFL. For non-highly myopic glaucoma, no significant differences were observed between pVD and pRNFL at any stage, consistent with findings by Yarmohammadi et al. (30) These results suggest that pVD is particularly valuable for early-stage glaucoma diagnosis, but its reliability diminishes in advanced disease stages.

## Limitations

This study had several limitations, which were systematically summarized as follows: First, the relatively small sample size, particularly for advanced glaucoma stages, required merging moderate and advanced stages in some analyses, which may limit generalizability. For example, only a small fraction of eyes were classified as advanced stage (MD < −12 dB), so separate analysis of advanced glaucoma was underpowered. This may reduce our ability to detect differences specific to advanced disease. Future studies including larger cohorts of advanced-stage patients are needed to validate these stage-specific findings. Second, geographical representativeness: The data were solely collected from two provinces, which might not have comprehensively reflected the characteristics of other populations. To enhance the validity of the findings, future research should have incorporated larger and more diverse cohorts. Third, technical limitations of the detection method (27): the SSADA algorithm is susceptible to projection artifacts from superficial vessels, which may appear as false flow signals in deeper layers of the optic nerve head. These artifacts, caused by moving shadows of blood cells, are indistinguishable from true decorrelation signals and may limit the precision of superficial versus deep layer analysis in OCTA-based assessments. Additionally, manual delineation of the optic disc boundary in highly myopic eyes was performed by a single observer without formal assessment of inter-or intra-observer variability. This may affect the reproducibility of segmentation-dependent measurements. In future studies, we plan to incorporate intraclass correlation coefficient (ICC) or Kappa statistics to better assess the consistency and reliability of manual segmentation.

## Conclusion

In summary, this study demonstrates that both pVD and pRNFL are valuable biomarkers for diagnosing and monitoring highly myopic glaucoma, with pVD offering stronger correlations with visual function and comparable diagnostic utility in early stages. The use of OCTA to assess pVD offers a reliable, non-invasive method for glaucoma diagnosis, complementing structural assessments like pRNFL. The combination of pVD and pRNFL measurements demonstrated strong diagnostic performance, particularly in early-stage highly myopic glaucoma (AUC: 0.905–0.934), suggesting its potential utility in early detection. Moreover, pVD serves as a valuable marker for assessing the severity of visual field damage in myopic glaucoma patients.

## Data Availability

The original contributions presented in the study are included in the article/Supplementary material, further inquiries can be directed to the corresponding author.
