# Supplementary material for: The role of peripapillary vessel density and retinal nerve fiber layer thickness in diagnosing and monitoring myopic glaucoma
Source: Front Med (Lausanne). 2025 Jun 16;12:1620968. doi: 10.3389/fmed.2025.1620968 (PMC12206875; doi:10.3389/fmed.2025.1620968)
Supplement: Supplementary file 1 [file Data_Sheet_1.docx]

Table S1. Comparison of the diagnostic capabilities of the pRNFL in 8 regions for highly myopic glaucoma

| Regions | Temporal superior (2) | Temporal inferior (3) | Inferior temporal (4) | Inferior nasal (5) | Nasal inferior (6) | Nasal superior (7) | Superior nasal (8) |
| --- | --- | --- | --- | --- | --- | --- | --- |
| Superior temporal (1) | 1>2(P<0.001) | 1>3(P=0.004) | 1=4(P=0.928) | 1=5(P=0.018) | 1>6(P<0.001) | 1>7(P<0.001) | 1>8(P<0.001) |
| Temporal superior (2) |  | 2=3(P=0.763) | 2<4(P=0.002) | 2=5(P=0.855) | 2>6(P<0.001) | 2>7(P<0.001) | 2=8(P=0.279) |
| Temporal inferior (3) |  |  | 3<4(P=0.001) | 3=5(P=0.983) | 3>6(P<0.001) | 3>7(P<0.001) | 3=8(P=0.182) |
| Inferior temporal (4) |  |  |  | 4>5(P=0.004) | 4>6(P<0.001) | 4>7(P<0.001) | 4>8(P<0.001) |
| Inferior nasal (5) |  |  |  |  | 5>6(P<0.001) | 5>7(P<0.001) | 5=8(P=0.163) |
| Nasal inferior (6) |  |  |  |  |  | 6=7(P=0.443) | 6<8(P<0.001) |
| Nasal superior (7) |  |  |  |  |  |  | 7<8(P<0.001) |

Bonferroni: p<0.00625. pRNFL, peripapillary retinal nerve fiber layer.

Table S2. Comparison of the diagnostic capabilities of the pVD in 8 regions for highly myopic glaucoma

| Regions | Temporal superior (2) | Temporal inferior (3) | Inferior temporal (4) | Inferior nasal (5) | Nasal inferior (6) | Nasal superior (7) | Superior nasal (8) |
| --- | --- | --- | --- | --- | --- | --- | --- |
| Superior temporal (1) | 1>2(P<0.001) | 1>3(P=0.001) | 1=2(P<0.623) | 1>5(P<0.001) | 1>6(P<0.001) | 1>7(P<0.001) | 1>8(P<0.001) |
| Temporal superior (2) |  | 2=3(P=0.111) | 2<4(P<0.001) | 2=5(P=0.574) | 2>6(P=0.004) | 2=7(P=0.017) | 2=8(P=0.925) |
| Temporal inferior (3) |  |  | 3<4(P=0.001) | 3=5(P=0.534) | 3>6(P<0.001) | 3>7(P=0.001) | 3=8(P=0.268) |
| Inferior temporal (4) |  |  |  | 4>5(P<0.001) | 4>6(P<0.001) | 4>7(P<0.001) | 4>8(P<0.001) |
| Inferior nasal (5) |  |  |  |  | 5>6(P<0.001) | 5>7(P=0.003) | 5=8(P=0.619) |
| Nasal inferior (6) |  |  |  |  |  | 6=7(P=0.309) | 6<8(P=0.001) |
| Nasal superior (7) |  |  |  |  |  |  | 7<8(P=0.004) |

Bonferroni: p<0.00625. pVD, peripapillary vessel density.

Table S3. Comparison of the diagnostic capabilities of the pRNFL in 8 regions for non-highly myopic glaucoma

| Regions | Temporal superior (2) | Temporal inferior (3) | Inferior temporal (4) | Inferior nasal (5) | Nasal inferior (6) | Nasal superior (7) | Superior nasal (8) |
| --- | --- | --- | --- | --- | --- | --- | --- |
| Superior temporal (1) | 1>2(P=0.002) | 1>3(P=0.003) | 1=4(P=0.383) | 1=5(P=0.907) | 1>6(P<0.001) | 1>7(P=0.002) | 1=8(P<0.257) |
| Temporal superior (2) |  | 2=3(P=0.508) | 2<4(P=0.001) | 2=5(P=0.020) | 2>6(P=0.118) | 2=7(P<0.949) | 2<8(P=0.001) |
| Temporal inferior (3) |  |  | 3<4(P<0.001) | 3=5(P=0.010) | 3=6(P=0.324) | 3=7(P=0.660) | 3<8(P<0.001) |
| Inferior temporal (4) |  |  |  | 4=5(P=0.320) | 4>6(P<0.001) | 4>7(P=0.002) | 4=8(P=0.995) |
| Inferior nasal (5) |  |  |  |  | 5>6(P<0.001) | 5>7(P=0.005) | 5=8(P=0.356) |
| Nasal inferior (6) |  |  |  |  |  | 6<7(P=0.005) | 6<8(P<0.001) |
| Nasal superior (7) |  |  |  |  |  |  | 7<8(P<0.001) |

Bonferroni: p<0.00625. pRNFL, peripapillary retinal nerve fiber layer.

Table S4. Comparison of the diagnostic capabilities of the pVD in 8 regions for non-highly myopic glaucoma

| Regions | Temporal superior (2) | Temporal inferior (3) | Inferior temporal (4) | Inferior nasal (5) | Nasal inferior (6) | Nasal superior (7) | Superior nasal (8) |
| --- | --- | --- | --- | --- | --- | --- | --- |
| Superior temporal (1) | 1=2(P=0.117) | 1=3(P=0.479) | 1=4(P<0.039) | 1=5(P<0.231) | 1=6(P=0.417) | 1=7(P<0.296) | 1=8(P<0.382) |
| Temporal superior (2) |  | 2=3(P=0.600) | 2<4(P<0.001) | 2=5(P=0.011) | 2=6(P=0.470) | 2=7(P=0.783) | 2=8(P=0.525) |
| Temporal inferior (3) |  |  | 3=4(P=0.008) | 3=5(P=0.097) | 3=6(P<0.935) | 3=7(P=0.815) | 3=8(P=0.953) |
| Inferior temporal (4) |  |  |  | 4=5(P<0.717) | 4>6(P<0.003) | 4>7(P=0.001) | 4=8(P<0.007) |
| Inferior nasal (5) |  |  |  |  | 5=6(P=0.046) | 5=7(P=0.022) | 5=8(P=0.081) |
| Nasal inferior (6) |  |  |  |  |  | 6=7(P=0.646) | 6=8(P=0.975) |
| Nasal superior (7) |  |  |  |  |  |  | 7=8(P=0.721) |

Bonferroni: p<0.00625. pVD, peripapillary vessel density.
